# A New Stud Farm in Hungary

**Published:** 1897-08

**Authors:** 


A New Stud Farm in Hungary.—The Imperial War Ministry has leased the estate Lábod, in Hungary, for twenty-five years. The estate embraces about eight thousand acres, and the price paid is 25,000 florins per year. The government will establish a stud farm there about October 1st.—*Their. Centralblatt*, November 15, 1896.

